# Mind the gap: identifying what is missed when searching only the broad scope with clinical queries

**DOI:** 10.5195/jmla.2019.589

**Published:** 2019-07-01

**Authors:** Edwin Vincent Sperr

**Affiliations:** Clinical Information Librarian, Office of Graduate Medical Education, Augusta University/University of Georgia Medical Partnership, Athens, GA, esperr@uga.edu

## Abstract

**Objective:**

The PubMed Clinical Study Category filters are subdivided into “Broad” and “Narrow” versions that are designed to maximize either sensitivity or specificity by using two different sets of keywords and Medical Subject Headings (MeSH). A searcher might assume that all items retrieved by Narrow would also be found by Broad, but there are occasions when some *[Filter name]*/Narrow citations are missed when using *[Filter name]*/Broad alone. This study quantifies the size of this effect.

**Methods:**

For each of the five Clinical Study Categories, PubMed was searched for citations matching the query *Filter*/Narrow NOT *Filter*/Broad. This number was compared with that for *Filter*/Broad to compute the number of Narrow citations missed per 1,000 Broad. This process was repeated for the MeSH terms for “Medicine” and “Diseases,” as well as for a set of individual test searches.

**Results:**

The Clinical Study Category filters for Etiology, Clinical Prediction Guides, Diagnosis, and Prognosis all showed notable numbers of *Filter*/Narrow citations that were missed when searching *Filter*/Broad alone. This was particularly true for Prognosis, where a searcher could easily miss one Prognosis/Narrow citation for every ten Prognosis/Broad citations retrieved.

**Conclusions:**

Users of the Clinical Study Category filters (except for Therapy) should consider combining *Filter*/Narrow together with *Filter*/Broad in their search strategy. This is particularly true when using Prognosis/Broad, as otherwise there is a substantial risk of missing potentially relevant citations.

## INTRODUCTION

It is widely appreciated that the rapid proliferation of the biomedical literature has made it increasingly difficult for health care practitioners to efficiently find the information that they need to do their jobs. In large bibliographic databases such as MEDLINE, the sorts of rigorous clinical studies that might inform clinical decision-making are mixed together with a host of letters, case reports, and notes from the frontiers of bench science. This poses a challenge to full-time clinicians, who typically have minimal training in bibliographic searching and very little time to devote to locating resources. Many measures have been designed to deal with this issue, including the use of specialized search “hedges” (or “filters”) for clinicians.

Search hedges are tools that are designed to focus a database search by emphasizing retrieval of items relating to a particular topic. These can be implemented in many ways, but most often take the form of pre-coordinated strings of text words, phrases, and subject headings that are combined with a user’s search to selectively limit retrieval. By ensuring that all results contain the words or concepts contained in the hedge, it is possible to increase the number of putatively relevant results for a given search, while simultaneously decreasing the effort required on the part of the searcher. Hedges are sometimes used by librarians when performing complex searches, but it is unlikely that non-information professionals would use these tools unless they were made explicitly aware of them. It is to this end that the National Library of Medicine has linked a tool called the “PubMed Clinical Queries” from the front page of PubMed [1].

The PubMed Clinical Queries tool dates back to the first iteration of PubMed in 1997 [2] and was based on the work of R. B. Haynes and his group of researchers at McMaster University [3]. Over the following years, Haynes et al. further refined their filters for “Therapy” [4], “Diagnosis” [5], “Etiology” [6], and “Prognosis” [7] and developed one for “Clinical prediction guides” [8]. These five filters still make up the heart of PubMed Clinical Queries. There, grouped together under “Clinical Study Categories,” they share space with filters for “Systematic Reviews” and “Medical Genetics.”

The development process for each of these five filters involved compiling candidate text words, phrases, and Medical Subject Headings (MeSH) and then using those to develop test strategies. These strategies were then each validated against a database of hand-selected “high-quality” articles that were chosen as exemplars of the sort of materials that are most helpful for clinicians. The best of these strategies were then further fine-tuned for performance along several axes, particularly sensitivity (“the proportion of high-quality articles retrieved”) and specificity (“how well the filter rejects low quality materials”) [5].

It is likely that different searchers would have dissimilar needs depending on the context or the topics of their searches. Therefore, separate variants of each filter were developed by Haynes et al. to balance sensitivity and specificity in different ways. Two of these iterations are included for each of the Clinical Study Categories: “Broad” and “Narrow.” Broad queries are tuned to emphasize sensitivity and are intended for “those interested in comprehensive retrievals or in searching for clinical topics with few citations” [5]. By contrast, the Narrow versions are optimized for specificity: “retrieval with little non-relevant material.” Table 1 shows exactly how this is implemented for the pair of queries that cover the concept of Prognosis [9].

**Table 1:** Clinical query for “Prognosis”

Filter	Sensitive/specific	Terms used
Prognosis: Broad	90%/80%	(incidence[MeSH:noexp] OR mortality[MeSH Terms] OR follow up studies[MeSH:noexp] OR prognos*[Text Word] OR predict*[Text Word] OR course*[Text Word])
Prognosis: Narrow	52%/94%	(prognos*[Title/Abstract] OR (first[Title/Abstract] AND episode[Title/Abstract]) OR cohort[Title/Abstract])

**Figure 1:**
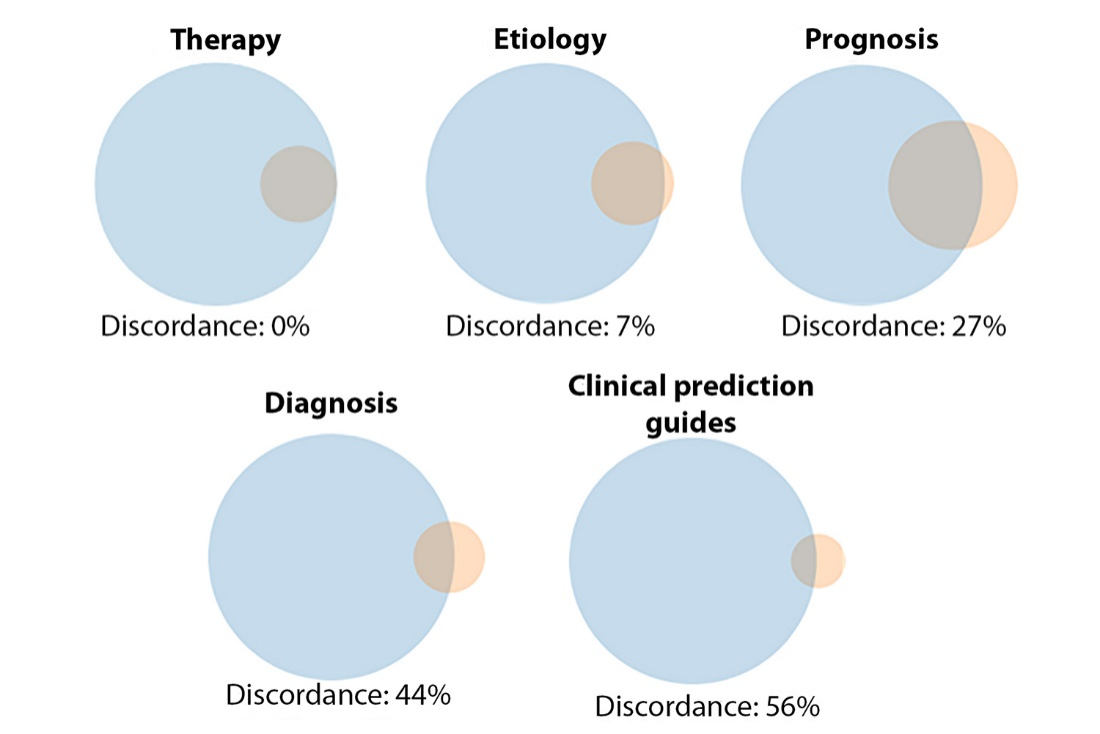
Discordances between *Filter*/Narrow and *Filter*/Broad for clinical study categories

It is important to note that the Broad and Narrow versions of each Clinical Study Category filter are not necessarily linked by terminology. Indeed, in some ways, the Clinical Study Categories comprise ten distinct hedges rather than five. This is understandable, as they were tested and optimized to meet two slightly different end points, but this difference in terms means that there is no guarantee that one would find all items of the set *[Filter name]*/Narrow (henceforth, *Filter*/Narrow) contained in the larger set *[Filter name]*/Broad (*Filter*/Broad). The filter descriptions, as well as the colloquial meanings of the terms “Broad” and “Narrow,” certainly imply that they would be, but comparing each set of filters with the interactive tool PubVenn shows that this is not necessarily the case [10] (Figure 1).

Searchers using *Filter*/Broad are likely looking for “comprehensive retrievals” that would include as many potentially relevant citations as can be easily identified though the Clinical Study Category tools. Citations selected by *Filter*/Narrow can be presumed to be at least as relevant to users’ searches as those selected by *Filter*/Broad, yet in many cases there is some portion of those citations that *Filter*/Broad users will never see unless they change their search strategies. This study explores the potential significance of this effect by quantifying its size.

## METHODS

The significance of missing a given number of potentially relevant citations for a search depends in part on the size of that search’s total result set. In this case, one would compare the number of citations selected by *Filter/*Broad to the size of that set of citations missed by *Filter/*Broad but retrieved by *Filter*/Narrow. It is possible to calculate the number of *Filter*/Narrow citations missed per 1,000 *Filter*/Broad citations with the following formula:

$$\left(\frac{\begin{array}{c} number\;of\;citations\;found\;by\;“Filter\\ name/\text{Narrow}[\text{filter}]\;\text{NOT }Filter\;name/\text{Broad}[\text{filter}]” \end{array}}{\begin{array}{c} number\;of\;citations\;found\;by“Filter\\ name/\text{Broad}[\text{filter}]” \end{array}}\right)\times 1,000$$

It is a straightforward task to calculate the above number for each of the five Clinical Study Categories. For example, the search “Therapy/Narrow[filter] NOT Therapy/Broad[filter]” yields zero results. This indicates that a user of Therapy/Broad could, uniquely among the Clinical Study Categories, always expect to find all members of Therapy/Narrow in their results. By contrast, “Diagnosis/Narrow[filter] NOT Diagnosis/Broad[filter]” yields 183,874 results (as of October 2018). Comparing this to the results of “Diagnosis/Broad[filter]” (4,983,419) gives 37 Narrow citations missed per 1,000 Broad retrieved.

One must be careful, however, about generalizing from the results of searches in the entirety of PubMed (henceforth, All PubMed). While the focus of PubMed is human clinical medicine, it also includes citations about nursing, veterinary science, bench research, and more. Therefore, it is possible that the significance of discordances between *Filter*/Narrow and *Filter*/Broad for searches about medical topics differs from those for those in All PubMed.

One way of addressing this question is to isolate and examine subsets of medicine-related citations. The calculations outlined above could be repeated but by combining each filter with the MeSH term “Medicine.” This would yield numbers of *Filter*/Narrow items missed in a set of citations specifically classified as pertaining to the “art and science of studying, performing research on, preventing, diagnosing, and treating disease” [11]. Similarly, it could be useful to investigate those citations indexed under “Diseases category” [MeSH terms], as it is a reasonable assumption that Clinical Study Category filter users will employ them while researching a particular disease [12].

The default behavior in PubMed is for subject searches to be “exploded” so that searches for broader subject headings also retrieve those citations indexed by the subjects that are “underneath” them in the tree structure. Therefore, these two searches incorporate results for many specific branches of medicine and individual diseases as well.

It is also important to note that it is entirely possible that discordances observed in the large citation aggregates described above might differ from those seen in the context of an individual search. To model real-world conditions, a list of individual sample searches was created, and the number of Narrow citations missed per 1,000 Broad was calculated for each search. These 209 simple searches were derived from a publicly available list of common International Statistical Classification of Diseases and Related Health Problems, 10th revision (ICD-10) codes [13]. Table 2 lists examples of test searches. The test searches were specifically formulated to cover a broad range of clinical concerns so that some of the searches would retrieve relatively large citation sets (e.g. “heart disease”), while others would be more specific (“Vitamin D Deficiency Anemia”).

**Table 2:** Examples of test searches

abdominal pain
Acute Kidney Failure
AIDS
Arthritis
Atopic Dermatitis
bone cancer
Cellulitis
Chronic Kidney Disease
Coronary Artery Disease
Diabetes
Dysmenorrhea
Edema
Flatulence
Gastroenteritis
heart disease
HPV
Monoclonal Gammopathy
Neuropathy
Osteoarthritis
Overweight[Mesh:NoExp]
Peripheral Vascular Disease
Polyuria
sexually transmitted disease
Syncope
Transient Ischemic Attack
Vitamin D Deficiency Anemia
weight loss

It would be challenging to perform so many searches manually without introducing errors of transcription into the results. For this reason, the author took advantage of the National Center for Biotechnology Information (NCBI) E-utilities application programming interface (API), which enables users to programmatically perform searches in NCBI databases using the framework of their choice [14]. A small Python program was written to iterate through each query in the test search set and then search it against PubMed. Results were returned by the API for both *Filter*/Broad and *Filter*/Narrow NOT *Filter*/Broad, and the program then calculated the number missed in each case (script and full list of test searches are available from the author’s Mind the Gap site https://osf.io/r97db/). The data resulting from the test searches were analyzed using Microsoft Excel.

## RESULTS

Data were most recently gathered and analyzed in October 2018 and are summarized in Table 3. As noted above, all results for Therapy/Narrow are found in Therapy/Broad, meaning that there are no Therapy/Narrow results missed when using the latter. Therefore, this analysis will be restricted to the remaining four Clinical Study Categories.

**Table 3:** *Filter*/Narrow citations missed per 1,000 *Filter*/Broad when searching Broad alone

Filter	All PubMed	Diseases	Medicine	Test queries		

				Median	Interquartile range	Range
Etiology	9	6	8	8	6–11	0–21
Clinical prediction guides	27	20	45	14	14–19	2–38
Diagnosis	37	12	9	5	3–11	0–43
Prognosis	78	75	82	100	66–138	20–387

Of those four categories, Etiology consistently had the fewest Narrow citations missed when searching Broad. For Etiology, results seen for “Medicine,” “Diseases,” and the test queries were similar to those for All PubMed. Clinical Prediction Guides and Diagnosis were close together in terms of results for All PubMed but showed divergent behavior otherwise. The number of “Medicine” Narrow citations missed for Clinical Prediction Guides was notably higher than in All PubMed, but the median for the test search set was considerably lower. For Diagnosis, numbers missed for both aggregates of citations and the median for the test search set were all much lower than for All PubMed. Indeed, the median number missed for the test search set was approximately one seventh of that seen in the latter.

**Figure 2:**
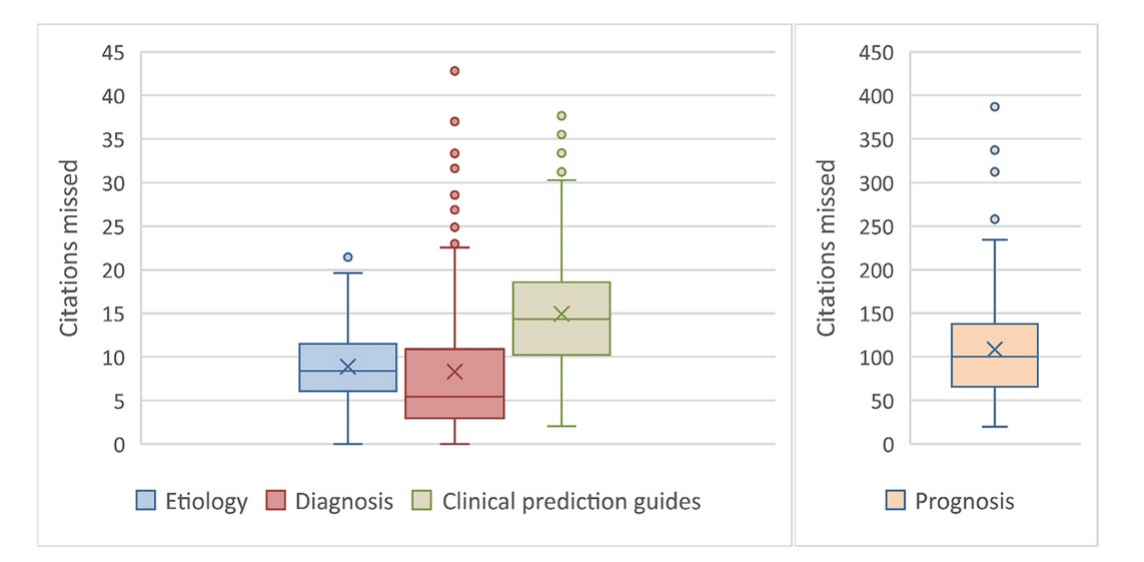
*Filter*/Narrow citations missed per 1,000 *Filter*/Broad in test queries

Prognosis is the outlier of the categories, with a higher number missed in All PubMed than any of the other filters, and notably more citations missed in the aggregates as well. The discrepancy is most dramatic in the results for the Prognosis test queries, where the median value of 100 Narrow citations missed per 1,000 Broad dwarfs that seen in the other 3 filters. Figure 2 illustrates the distribution of values of Narrow citations missed per 1,000 Broad for the 4 sets of test queries.

### Comparing Narrow NOT Broad citations to Narrow AND Broad citations

**Figure 3:**
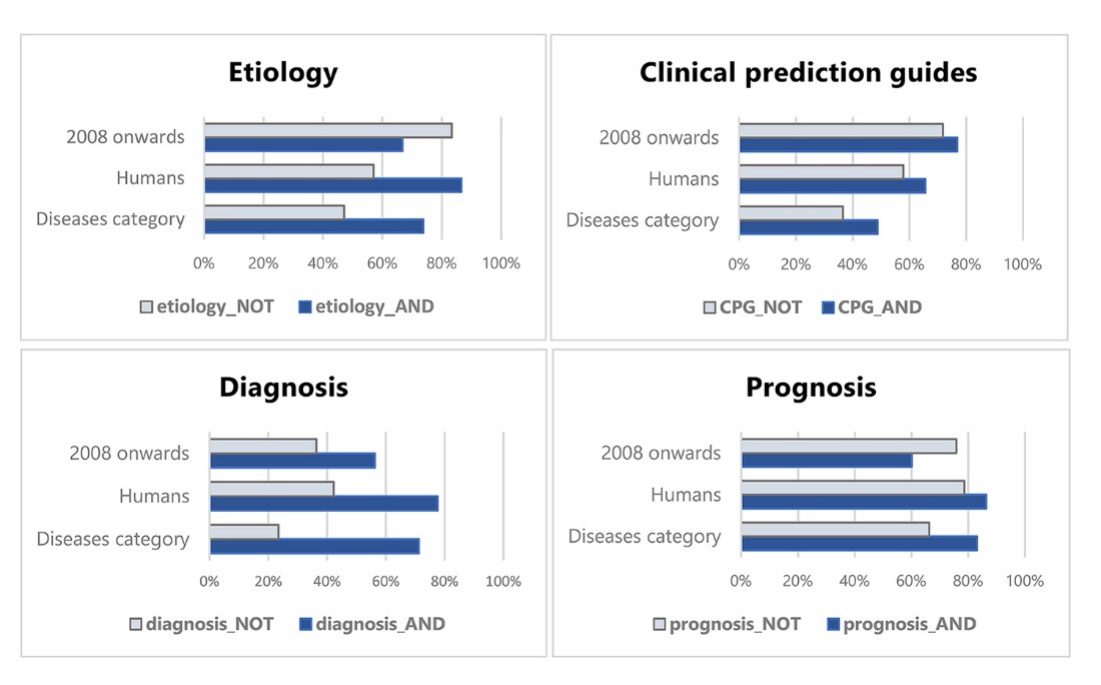
Proportion of citations matching selected criteria for each part of *Filter*/Narrow

As demonstrated above, it is simple to isolate those Narrow citations that are included by *Filter*/Narrow but rejected by *Filter*/Broad using the query “*[Filter name]*/Narrow[filter] NOT *[Filter name]*/Broad[filter].” Similarly, one can select the set of Narrow citations that are selected by both with the query “*[Filter name]*/Narrow[filter] AND *[Filter name]*/Broad[filter].” Once both subparts of *Filter*/Narrow have been so defined, it is possible to search each set in PubMed against terms that might differentiate them in meaningful ways. Figure 3 compares *Filter*/Narrow NOT *Filter*/Broad citations to *Filter*/Narrow AND *Filter*/Broad citations, according to what proportion of each set is indexed by the subject headings “Diseases Category” or “Humans,” as well as whether it has a publication date of 2008 or later.

For each filter, a smaller proportion of Narrow NOT Broad citations were indexed with “Diseases Category” or “Humans” than Narrow AND Broad, though this difference varied between filters. Interestingly, Narrow NOT Broad citations for Etiology and Prognosis were a bit more likely to be recent citations than their Narrow AND Broad counterparts.

## DISCUSSION

While any database filter is approximate in its effect, both the Broad and Narrow iterations of the Clinical Study Categories are designed to maximize the amount of clinically relevant material for a given search. For each Category, Haynes et al. carefully tuned the Narrow version to emphasize “retrieval with little non-relevant material.” Therefore, it seems safe to posit that the citations selected by *Filter*/Narrow for a given search should (at least on balance) be just as relevant as those selected by *Filter*/Broad, if not more so.

This study has revealed that there is a consistent pattern where some citations selected by the Narrow versions of four out of the five Clinical Study Category filters are simultaneously rejected by their Broad counterparts. It has been further demonstrated that this effect appears consistently for All PubMed, across the “Medicine” and “Diseases” citation aggregates, and in a broad cross section of the types of searches that filter users are likely to perform.

This effect does vary in intensity. A user of Diagnosis/Broad alone is likely to miss at least 5 putatively relevant Narrow citations for each 1,000 retrieved; using Etiology/Broad that number would probably climb to 8. This effect becomes particularly pronounced in the case of Prognosis, as a user of Prognosis/Broad alone would likely miss ten times as many.

Individual searches can show even larger discordances. Many of the Etiology/Broad test searches showed 10 Narrow citations missed for every 100 Broad citations retrieved, and a search using Diagnosis/Broad could easily miss twice that number. Nearly half of the Prognosis/Broad test searches showed 1 Narrow citation missed for every *10* retrieved, and in one case (“gestational diabetes”), that number climbed to nearly 4 in 10. Without manually checking, users have no way of knowing where on this continuum their searches might lie.

The differences noted above between Narrow NOT Broad citations and Narrow AND Broad citations illuminate useful avenues of future study. In particular, it is notable that Narrow NOT Broad citations are generally less likely to be indexed with “Diseases category” [Mesh] or “Humans” [Mesh] than their Narrow AND Broad counterparts, a fact that could potentially be taken as an indicator of lesser clinical relevance. However, such differences do not diminish the importance of the phenomenon of *Filter*/Narrow citations being excluded by *Filter*/Broad. For one thing, Narrow NOT Broad citations make up a large proportion of all Narrow citations in three of the five Clinical Study Categories. One could not dismiss their relevance without calling the effectiveness of *Filter*/Narrow itself into question. More to the point, an examination of individual *Filter*/Narrow NOT *Filter*/Broad citations reveals that many of them are indeed clinically “relevant,” according to the criteria set forth by the Haynes et al. group. Indeed, many Prognosis/Narrow NOT Prognosis/Broad citations appear in the hand-curated McMaster Plus database, the primary test criteria used in the 2013 revalidation study of the Clinical Study Category filters by Wilczynski et al. [15, 16].

The importance of these missing citations is to some degree dependent on the task at hand. If one is searching for a couple of reviews of a common condition using Diagnosis/Broad, it might make little difference if 1 or 2 putatively relevant citations out of 100 are missed. If one is attempting to conduct a more thorough review of the literature instead, that relatively small number of missed citations could well be significant. By contrast, a user of Prognosis/Broad for most any search task would likely be concerned, because there is a real prospect of missing 1 potentially relevant result for every 10 retrieved.

The data shown above demonstrate that PubMed users should be aware of the behavior of the Clinical Study Categories when using them to limit their retrievals. For Etiology, Diagnosis, Clinical Prediction Guides, and Prognosis, many putatively relevant *Filter*/Narrow citations are missed when searching *Filter*/Broad alone. If they are interested in retrieving as much relevant material as possible, these users should consider the expedient of using a Boolean OR to combine *Filter*/Broad with *Filter*/Narrow as part of their search strategy. This is especially the case when using Prognosis/Broad, because otherwise, there is a substantial risk of missing relevant citations.
